# Poincaré Plot Nonextensive Distribution Entropy: A New Method for Electroencephalography (EEG) Time Series

**DOI:** 10.3390/s22166283

**Published:** 2022-08-21

**Authors:** Xiaobi Chen, Guanghua Xu, Chenghang Du, Sicong Zhang, Xun Zhang, Zhicheng Teng

**Affiliations:** 1 School of Mechanical Engineering, Xi’an Jiaotong University, Xi’an 710049, China; 2 State Key Laboratory for Manufacturing Systems Engineering, Xi’an Jiaotong University, Xi’an 710049, China

**Keywords:** nonextensive, distribution entropy, Poincaré plot, sector ring subinterval

## Abstract

As a novel form of visual analysis technique, the Poincaré plot has been used to identify correlation patterns in time series that cannot be detected using traditional analysis methods. In this work, based on the nonextensive of EEG, Poincaré plot nonextensive distribution entropy (NDE) is proposed to solve the problem of insufficient discrimination ability of Poincaré plot distribution entropy (DE) in analyzing fractional Brownian motion time series with different Hurst indices. More specifically, firstly, the reasons for the failure of Poincaré plot DE in the analysis of fractional Brownian motion are analyzed; secondly, in view of the nonextensive of EEG, a nonextensive parameter, the distance between sector ring subintervals from the original point, is introduced to highlight the different roles of each sector ring subinterval in the system. To demonstrate the usefulness of this method, the simulated time series of the fractional Brownian motion with different Hurst indices were analyzed using Poincaré plot NDE, and the process of determining the relevant parameters was further explained. Furthermore, the published sleep EEG dataset was analyzed, and the results showed that the Poincaré plot NDE can effectively reflect different sleep stages. The obtained results for the two classes of time series demonstrate that the Poincaré plot NDE provides a prospective tool for single-channel EEG time series analysis.

## 1. Introduction

Discussing the complexity of time series has long been a challenge, and researchers have conducted a lot of work in this regard. Among them, entropy plays an important role in measuring the complexity of time series. In order to meet different experimental requirements and obtain better results, several concepts came into being: sample entropy, transfer entropy, permutation entropy, and fuzzy entropy [1,2,3,4]. Most of the previous entropy analysis is based on Shannon entropy [5], which has undeniable shortcomings in dealing with some specific problems. For this reason, more accurate and robust analysis techniques are being developed to make the analyzed signal less susceptible to noise and artifacts in the signal, which is currently an active research area. Because of its intuitive expression, strong anti-noise ability, and low requirements for data length and stability, the Poincaré plot has attracted more and more attention [6,7], especially in the analysis of physiological signals with obvious nonstationary and nonlinear characteristics. The Poincaré plot is a geometric representation method that maps time series to a Cartesian plane, and previous studies posited that it can display various nonlinear dynamic patterns of signals [8]. The coordinates of scattered points in the plot are represented by two pairs of points in the original sequence. The time interval between two points is the delay of the Poincaré plot. When the delay is 1, its standard form is obtained, showing the correlation between adjacent points in the time series [9,10,11]. The traditional quantitative analysis method of the Poincaré plot mainly extracts mathematical features from its geometric distribution shape. Researchers have put forward some indices, such as long and short axis, width, area, vector index, complex correlation measure (CCM), etc. Originally, the Poincaré plot was widely used for the analysis of heart rate variability (HRV), focusing on the analysis of beat-to-beat fluctuations in heart rate and the diagnostic power these fluctuations provide [7,12,13,14]. As a typical physiological signal with high intensity, ECG signal has obvious changes in the amplitude or time interval of its related feature points under different physiological and pathological states, so that the Poincaré plot theoretical indices can be accurately associated with physiological causes, and satisfactory results have been achieved in previous studies. In view of the results obtained by the Poincaré plot in HRV research, some scholars have tried to use Poincaré plots to achieve quantitative assessment of different brain states by analyzing EEG [15,16,17,18,19]. In the above study, it was considered that the distribution of the Poincaré plot of EEG comprised ellipses. However, in fact, this was not always the case in the real EEG Poincaré plot distribution. Forcibly fitting the distribution of points will inevitably bring analysis errors and affect the reliability of analysis results. In addition, the ellipse fitting method mainly reflected the linear nature of the EEG, but cannot quantify the nonlinear information contained in it. Therefore, other scholars began to study the quantitative analysis methods and indices that can reflect the nonlinear characteristics of the Poincaré plot so as to make better use of the Poincaré plot to quantitatively analyze the nonlinear dynamic information of the signal. Huo et al. first proposed the concept of Poincaré plot distribution entropy (DE), which connected entropy with Poincaré plot analysis, and achieved good results in the analysis of HRV [20]. In this approach, concentric circles were used to divide the set of scattered points into multiple sector ring subintervals, and the scattered points in each sector ring subinterval were counted to analyze the distribution of scattered points. Higher DE indicated that the distribution of scattered points was more uniform. On the contrary, lower DE reflected that the points in the scatter diagram tend to concentrate in some sub regions. For ECG signals with obvious waveform changes in the time domain, Huo’s research conclusion is valid because it is not necessary to consider the influence of the distance of the scatter from the origin on the distribution entropy results. When his method is used to analyze EEG with insignificant changes in time domain waveforms in different states, the Poincaré plot DE will encounter the problem of insufficient discrimination. In order to obtain more general results, we further improved the Poincaré plot DE. Based on the nonextensive EEG [21,22,23], we not only considered the impact of the number of scatter points in each sector ring subinterval on the system, but also the impact of the distance between each sector ring subinterval and the origin on the system, and take the distance as a nonextensive parameter to give corresponding weights to each sector ring subinterval. Finally, based on the form of nonextensive entropy, the influence of each sector ring subinterval on the system is integrated macroscopically, and the Poincaré plot nonextensive distribution entropy (NDE) is proposed. In this work, we first used the Poincaré plot DE to analyze the simulated time series of fractional Brownian motion with sequentially increasing Hurst indices, and the results of the Poincaré plot DE calculations were analyzed. Secondly, the failure reason of Poincaré plot DE in analyzing the simulated time series of fractional Brownian motion was analyzed, and the calculation form of Poincaré plot DE was improved by introducing nonextensive parameters, highlighting the different effects of each sector ring subinterval on the system, and the Poincaré plot NDE was proposed. Next, the simulated time series of the same fractional Brownian motion were analyzed using Poincaré plot NDE, and the influence of the number of sector ring subintervals on the analysis results was discussed. Finally, after the validity of the Poincaré plot NDE was verified by using fractional Brownian motion data, the published sleep EEG dataset was analyzed, and the results showed that the Poincaré plot NDE can effectively reflect different sleep stages.

The reminder of this paper is organized as follows. In Section 2, the concept of Poincaré plot DE is introduced, and the time series of fractional Brownian motion with deferent Hurst indices increasing continuously are analyzed by Poincaré plot DE. According to the analysis results, the deficiency of distribution entropy of Poincare scattered points is pointed out, and the Poincaré plot NDE is proposed. In Section 3, the Poincaré plot NDE is applied to the fractional Brownian motion time series with continuously increasing Hurst indices and the open sleep EEG dataset. The determination process of relevant parameters was discussed and the analysis results were given. Finally, the conclusion was provided in Section 4.

## 2. Materials and Methods

### 2.1. Poincaré Plot DE

**Figure 1:**
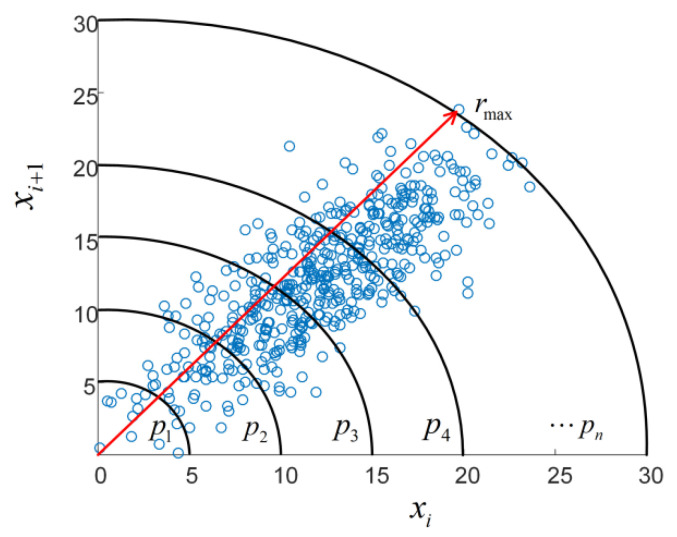
Schematic diagram of Poincaré plot sector ring subintervals. The blue ones are scatter points, the black ones are concentric arcs and the red rays are the maximum distance from the scattered point to the origin.

In order to reflect the properties of Poincaré plot in a more comprehensive and detailed way, Huo et al. proposed the Poincaré plot DE for the first time when analyzing HRV [21], which reflects the probability distribution characteristics of scattergram in the sector ring subinterval. In order to simplify the analysis, the time series is transformed so that all scatter points fall into the first quadrant of Cartesian coordinates. The realization process of distribution entropy is as follows:

1. For a time series $s_{i}(i=1\cdots N)$, calculate the minimum value $s_{\min}$ and $s_{i}(i=1\cdots N)$ subtract the minimum value $s_{\min}$ to obtain the time series of positive values $x_{i}(i=1\cdots N)$.
2. Poincaré plot is constructed with coordinates $\left\{(x_{i},x_{i+1}),(i=1\cdots N-1)\right\}$. $x_{i}$ is the abscissa and $x_{i+1}$ is the ordinate.
3. Take the origin as the center, make n concentric circles, and divide the quadrant into n sub regions of sector ring subintervals with equal width. The schematic diagram is shown in Figure 1. The radius of the *k*-th circle is:
  (1) $$r_{k}=r_{\max}/n*k,(1\leq k\leq n)$$
  where $r_{\max}$ is the maximum distance from the scatter point to the origin.
4. Count the number of scatter points $S_{num}^{k}$ in each sector ring subinterval, calculate its percentage in the total number N−1 of scatter points, and obtain the discrete probability set of scatter points partition: $p_{k}=S_{num}^{k}/(N-1)$. According to the definition of Shannon entropy, the Poincaré plot DE is:
  (2) $$E_{D}=-\sum_{k=1}^{n}p_{k}\times \log(p_{k})$$

The value of the Poincaré plot DE reflects the structural state of the distribution of scattered points. High distribution entropy means that the scatter points are evenly distributed in each sector ring subintervals. On the contrary, low distribution entropy means that the scattered points tend to concentrate in some sector ring subintervals.

### 2.2. Analysis of Poincaré Plot DE in Fractional Brownian Motion (FBM)

FBM provides useful models for many physiological phenomena that exhibit long-term dependence and 1/f spectral behavior. For EEG, its fractional behavior and abnormal power spectrum can be characterized by fractal dimension, power law index, and Hurst index. This is a simple mathematical model to describe EEG, which has been widely recognized in previous studies [24,25,26].

The fractional Brownian motion is a self-similar centralized Gaussian process $B^{H}={\left(B_{t}^{H}\right)}_{t>0}$, where *H* is Hurst index its covariance function is defined as:

(3) $$E[B_{t}^{H}B_{s}^{H}]=1/2(t^{2H}+s^{2H}-{\left|t-s\right|}^{2H})$$

When Hurst indices *H* are different, the system will show different motion patterns; *H* = 0.5 corresponds to typical Brownian motion. When *H* > 0.5, the system shows long-range positive correlation; it has long-range negative correlation for *H <* 0.5 [27].

**Figure 2:**
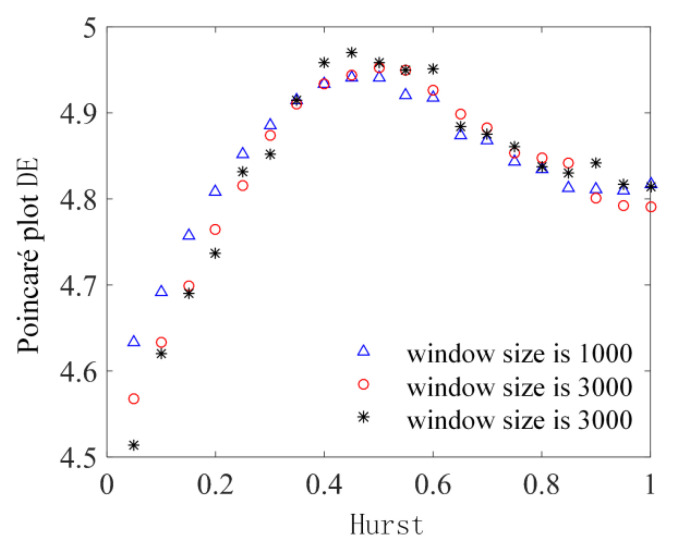
Poincaré plot DE analysis results of fractional Brownian motion time series with different Hurst indices when the window lengths are 1000, 3000, and 5000.

The original analysis object of Poincaré plot DE is ECG time series. In order to verify its effectiveness in EEG time series analysis, as EEG simulation data, fractional Brownian motion time series with different Hurst indices are analyzed. The time series of fractional Brownian motion is generated by the wfbm function of MATLAB. The length of the time series is 20,000 data points, and the Hurst index is evenly taken between (0, 1], with an interval of 0.05. To enhance the analysis, the generated time series are analyzed separately using sliding temporal windows with overlap between adjacent windows, a step size of 500 data points for the sliding time windows, and a sector ring partition size taken as 40. The data within each window are analyzed using the Poincaré plot DE, and for a time series, the average value of all sliding windows is taken as the measured value. When the window size is 1000, 3000, and 5000 data points, the analysis results of Poincaré plot DE of fractional Brownian motion time series with different Hurst indices are shown in Figure 2.

As shown in Figure 2, under the three window widths, the analysis results show the same trend. When the Hurst indices are less than 0.5, the values of the Poincaré plot DE shows an upward trend; when the Hurst indices are greater than 0.5, it shows a downward trend, and the change rate in the rising stage is greater than that in the falling stage. When the Hurst index approaches 1, the results tend to be stable. Existing studies have shown that the entropy of fractional Brownian motion decreases with the increase in Hurst index [28,29,30].

**Figure 3:**
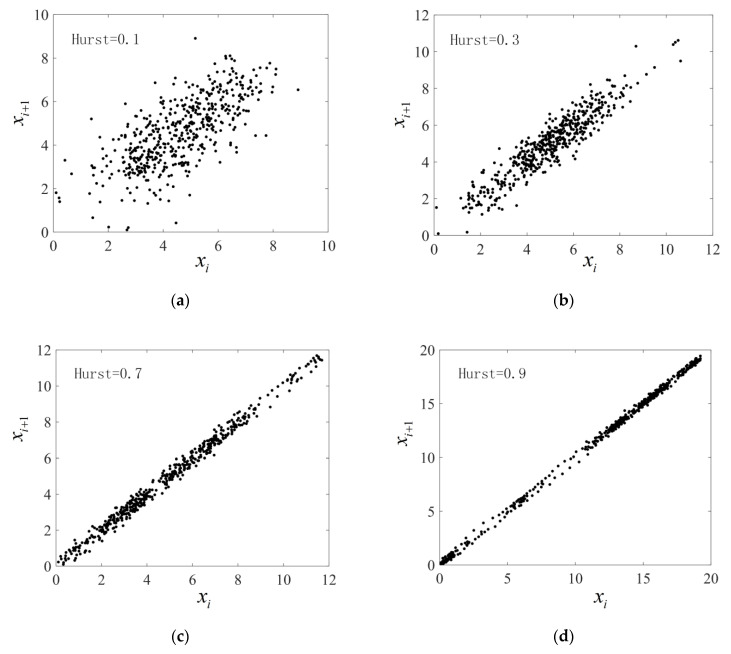
The Poincaré plots of fractional Brownian motion time series (**a**) when Hurst exponent is 0.1; (**b**) when Hurst exponent is 0.3; (**c**) when Hurst exponent is 0.7; (**d**) when Hurst exponent is 0.9.

The analysis results of Poincaré plot DE are obviously inconsistent with this conclusion, and there are even opposite conclusions within the range of individual Hurst indices. Thus, it is necessary to further investigate the reasons for the failure of Poincaré plot DE when analyzing fractional Brownian motion, because the results of Poincaré plot DE are mainly affected by the scatter distribution; thus, we selected the scatter plots of the time series of fractional Brownian motion at Hurst indices of 0.1, 0.3, 0.7, and 0.9 for analysis, and the length of the time series is 500 data points for identification purposes. The Poincaré plots of fractional Brownian motion time series when Hurst indices are 0.1, 0.3, 0.7, and 0.9 are shown in Figure 3a–d.

As an overall observation, with the increase in Hurst indices, the scattering point gradually approaches the 45° diagonal, and the maximum distance $r_{\max}$ from the farthest scattering point to the origin gradually increases. By comparing Figure 3a,b, when Hurst indices are less than 0.5, the scattered points with Hurst index of 0.1 are more scattered, but an obvious aggregation phenomenon can be observed, and the number of isolated scattered points increases. When the Hurst index is 0.3, the distribution range of the scatter points shrinks to the 45° diagonal with a more uniform distribution and a decrease in the number of isolated scatters. This distribution trend causes the Poincaré plot DE to increase with the increase in Hurst index in this range. Comparing Figure 3c,d, when the Hurst index is greater than 0.5, the larger the Hurst index, the more obvious the phenomenon of scattered points aggregation. When the Hurst index is 0.7, the aggregated scattered points are mainly distributed near the origin. When the Hurst index is 0.9, the aggregated scattered points are mainly distributed far away from the origin. This distribution form causes the results of Poincaré plot DE analysis to decrease with the increase in Hurst index within this range.

Based on the principle of Poincaré plot DE, it can be found that this method only considers the probability distribution characteristics of the scatter points in each sector ring subinterval, and does not consider the impact of the distance between the scatter points in each sector ring subinterval and the origin on the analysis results. For example, for a Poincaré plot A with five sector ring subintervals, according to the order of the distance between each subinterval and the origin, its discrete probability density set is {1/15, 2/15, 1/5, 4/15, 1/3}. For another Poincaré plot B with five sector ring subintervals, its discrete probability density set is {1/15, 2/15, 1/5, 4/15, 1/3}, according to the order from each subinterval to the origin. The Poincaré plot DE of these two Poincaré plot is the same, but it is obvious that the Poincaré plot distribution on the plane is completely different. Therefore, in the following analysis, the distance between the sector ring subinterval and the origin is introduced into the analysis of Poincaré plot as a parameter to measure the distribution state change more comprehensively.

### 2.3. Poincaré Plot Nonextensive Distribution Entropy (NDE)

In order to solve the shortage of the Poincaré plot DE, based on the concept of nonextensive distribution, the nonextensive parameter set is introduced to assign different weights to each sector ring subinterval, and then the influence of each sector ring subinterval on the whole is integrated in the form of nonextensive entropy. Finally, the concept of Poincaré plot NDE is proposed.

When there are long-range correlations, long-term memory effects, or fractal or multifractal dimensions, the system is considered to be nonextensive, such as self-gravitating systems, cosmology, nonlinear dynamic system, and so on [22,31]. We changed the calculation method so that the entropy is directly related to all the microscopic states of the system, and can provide a satisfactory physical explanation for the non-additive systems with hybrid or irregular fragment shapes. It is defined as:

(4) $$S_{q}=\frac{1-\sum_{i=1}^{W}p_{i}{}^{q}}{q-1}$$

where $p_{i}$ is the discrete probability, q is the nonextensive parameter, and W is the number of discrete probabilities. When q→1, the value of Shannon entropy is obtained by using the limit calculated by Lobita’s law:

(5) $$\underset{q\to 1}{\lim}S_{q}=\underset{q\to 1}{\lim}\frac{1-\sum_{i=1}^{W}p_{i}{}^{q}}{q-1}=-\sum_{i=1}^{W}p_{i}\log p_{i}$$

On the premise that the nonextensive of the system is uniform, q is used to describe the nonextensive distribution of the interaction between all constituent units in the system. When q<1, all structural units are mutually reinforcing; when q>1, all structural units are mutually inhibiting. This is not the case in an actual system. Structural units that promote or inhibit each other coexist in the system, and different structural units have different effects on the system. Therefore, different nonextensive parameters need to be used to describe the respective effects of structural units on the system as a whole. For this reason, Equation (4) is improved, and the nonextensive parameter q is extended to nonextensive parameter set: $q_{s}=\{q_{1},q_{2},q_{3},\cdots q_{i}\cdots q_{W}\}$, According to the literature of Tong [22]:

(6) $$p_{i}^{q_{i}-1}=e^{\left(q_{i}-1)\ln(p_{i}\right)}$$

According to the approximation formula $e^{x}\approx 1+x,(\left|x\right|\to 0)$, so when $q_{i}\to 1$

(7) $$e^{\left(q_{i}-1)\ln(p_{i}\right)}\approx 1+(q_{i}-1)\log(p_{i})$$

From Equations (6) and (7), we have

(8) $$p_{i}^{q_{i}-1}=1+(q_{i}-1)\log(p_{i})\Rightarrow \log(p_{i})=-(p_{i}^{q_{i}-1}-1)/(1-q_{i})$$

Input Equation (8) into Equation (5), and we can obtain:

(9) $$\underset{q\to 1}{\lim}S_{q}=-\sum_{i=1}^{W}p_{i}(p_{i}^{q_{i}-1}-1)/(1-q_{i})=-\sum_{i=1}^{W}(p_{i}{}^{q^{i}}-p_{i})/(1-q_{i})$$

**Figure 4:**
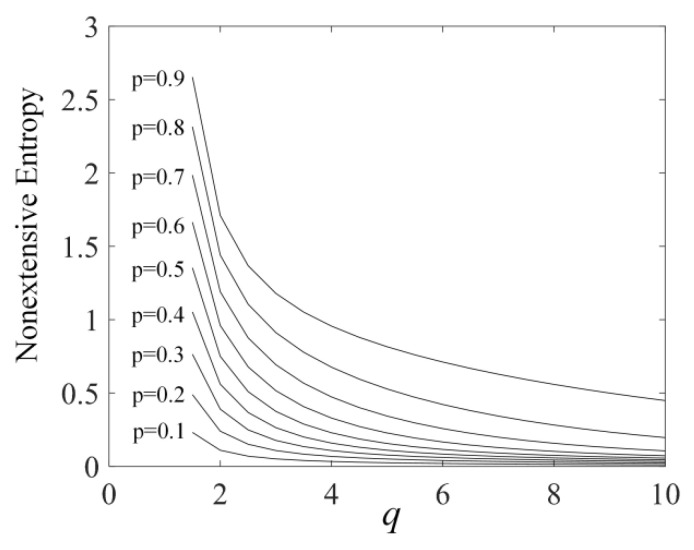
The corresponding relationship between the NE and the nonextensive parameter q when probability p takes different values.

Thus, a new form of entropy—new nonextensive entropy(NNE), which describes the different effects of each structural unit on the system—can be obtained. The expression form of NNE is obtained under the condition of q→1. Here, we further expand the application scope of this formula, relax the constraint condition to q>1, and only use the expression form of Equation (9) as a quantitative evaluation method for integrating the effects of various structural units on the system. To justify this expansion, the corresponding relationship between the value of NNE and the nonextensive parameter is calculated under different values of p. p is evenly valued between [0.1, 0.9] with an interval of 0.1, and q is evenly valued between (1, 10] with an interval of 0.5. The results are shown in Figure 4.

In Figure 4, when the nonextensive parameter q is greater than 1, the value of NE is positively correlated with the value of discrete probability p and negatively correlated with the value of non-extended parameter q. When the extended parameter *q* remains unchanged, the greater the value of discrete probability p, and the greater the contribution to the system. When the discrete probability p is fixed, the greater the value of non-extended parameter q, and the smaller the contribution to the system. Equation (9) perfectly integrates the influence of discrete probability p and nonextensive parameter q on the system. It can be seen that the expansion made to the constraints is reasonable.

Based on the above idea of NE, further improvements are made to the Poincaré plot DE. The first problem to be solved is the construction of discrete probability set and nonextensive parameter set. For the discrete probability set, the construction method of the discrete probability set in the Poincaré plot DE is adopted. For the construction of the nonextensive parameter set, considering the influence of the signal amplitude on the distribution of scattered points, the distance from each sector ring subinterval to the origin is introduced into the construction of the nonextensive parameter set, and each sector ring subinterval is assigned a nonextensive parameter related to the distance. This parameter needs to ensure that the sector ring subinterval close to the origin has little impact on the system and that the sector ring subinterval far from the origin has a great impact on the system, so it is reasonable to make such a provision, because when the scatter point is far from the origin, the signal amplitude is large, and when the scatter point is close to the origin, the signal amplitude is small. The realization process of Poincaré plot NDE is as follows:

1. For a time series $s_{i}(i=1\cdots N)$, calculate the minimum value $s_{\min}$ of s; subtract the minimum value $s_{\min}$ to obtain the positive time series $x_{i}(i=1\cdots N)$.
2. Construct a scatter plot with the scatter point pair $\left\{(x_{i},x_{i+1}),(i=1\cdots N-1)\right\}$ as the coordinates, where $x_{i}$ is the abscissa and $x_{i+1}$ is the ordinate.
3. Take the origin as the center, make W concentric circles, divide the quadrant into W sector ring subintervals, and the radius of the *k*-th circle is:
  (10) $$L_{k}=L_{\max}/W\times k,(1\leq k\leq W)$$
  where $L_{\max}$ is the maximum distance from each scatter point to the origin.
4. Count the number of scatter points in each sector ring subintervals $S_{num}^{k}$, and calculate the percentage of all N−1 scatter points to obtain the discrete probability set $\left\{p_{1},p_{2},\cdots p_{W}\right\}$ of the Poincaré plot.
5. The set of distances from the center point of each sector ring subinterval to the origin is taken as the nonextensive parameter set, and the nonextensive parameter set is $\left\{q_{1},q_{2},\cdot \cdot \cdot ,q_{W}\right\}$. Because the nonextensive parameters are negatively correlated with the NE, in order to satisfy the requirement that the sector ring subinterval far from the origin has a large impact on the system, the distance is processed by reverse subtraction, and the calculation process of the nonextensive parameters is as follows:
  (11) $$q_{k}=1+L_{\max}-[0:W-1]\times dl-dl/2$$
  where dl is the distance between rings of uniformly divided scatter plane.
6. Substitute the discrete probability set $\left\{p_{1},p_{2},\cdots p_{W}\right\}$ and the nonextensive parameter set $\left\{q_{1},q_{2},\cdot \cdot \cdot ,q_{W}\right\}$ into Equation (9) to calculate the entropy, and the obtained Poincaré plot NDE $S_{neq}$ is:
  (12) $$S_{neq}=-\sum_{k=1}^{W}(p_{k}{}^{q^{k}}-p_{k})/(1-q_{k})$$

The pseudo code for the Poincaré plot NDE algorithm is as follows (see Algorithm 1).
**Algorithm 1** Poincaré Plot NDEPoincaré plot NDE(W)          // W is the number of sector ring subintervals1  $s_{\min}=\min(s_{i})$           // calculate the minimum value of2  **for** i=1 **to**
N
3    $x_{i}=s_{i}-s_{\min}$          // to obtain the positive time series4  Construct scatter plot with $\left(x_{i},x_{i+1}\right)$5  **for** k=1 **to** W6    $L_{k}=L_{\max}/W\times k,(1\leq k\leq W)$ // radius of the concentric circle7  Divide the scatter plot into W sector ring subintervals8  **for** k=1 **to** W9    $p_{k}=S_{num}^{k}/(N-1)$           // to obtain the discrete probability sets10    $q_{k}=1+L_{\max}-(k-1)\times dl-dl/2$  // to obtain nonextensive parameter sets11   $S_{neq}=0$
12   **for**
k=1 **to** W
13   $S_{neq}=S_{neq}-(p_{k}{}^{q^{k}}-p_{k})/(1-q_{k})$14   **return**  $S_{neq}$


## 3. Data Analysis

### 3.1. Analysis for Fractional Brownian Motion (FBM)

**Figure 5:**
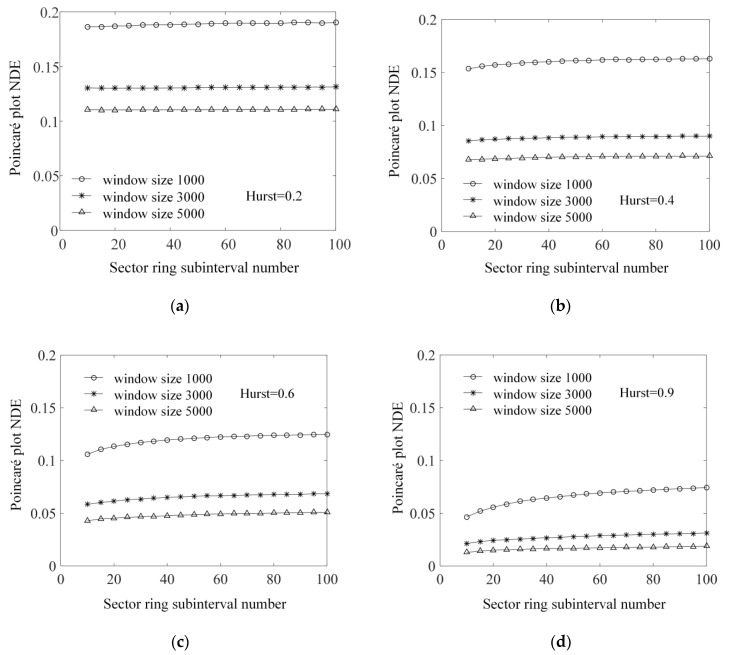
When the sliding time window widths are 1000, 3000, and 5000, the corresponding relationship between the Poincaré plot NDE of the fractional Brownian motion time series and the number of sector ring subintervals changes continuously. (**a**) Hurst index is 0.2; (**b**) Hurst index is 0.4; (**c**) Hurst index is 0.6; (**d**) Hurst index is 0.9.

To verify the validity of the Poincaré plot NDE method, time series of fractional Brownian motion with different Hurst indices are analyzed. The time series of fractional Brownian motion are generated by MATLAB’s wfbm function. The length of the time series is 20,000 data points and the Hurst indices are taken uniformly between (0, 1] with an interval of 0.05. The same sliding time window are calculated using Algorithm 1 for the fractional Brownian motion time series. The two parameters to be determined before starting the analysis are the window width and the number of sector ring subintervals. The number of sector ring subintervals is closely related to the Poincaré plot NDE results, and when the sector ring subinterval number changes, the Poincaré plot NDE value also changes. In order to study the law of change between sector ring subinterval number and the Poincaré plot NDE, the fractional Brownian motion time series with Hurst indices of 0.2, 0.4, 0.6, and 0.9 are selected for analysis. The sliding window method is used to calculate the Poincaré plot NDE of each time series when the number of sector ring subintervals changes from 10 to 100 in increments of 5. In order to suppress the influence of different window lengths on the analysis results, the sliding time window widths are taken as 1000, 3000, and 5000 data points. The average of all sliding window analysis results is used as the measurement value for the time series. The results obtained are shown in Figure 5.

The window width of sliding time window and the number of sector ring subintervals have an impact on the Poincaré plot NDE of fractional Brownian motion time series. Among them, the size of window width has a greater impact, and the larger the value of window width, the smaller the value of Poincaré plot NDE. When the Hurst index is small, as shown in Figure 5a,b, the change in the number of sector ring subintervals has little effect on the value of Poincaré plot NDE. When the Hurst index is 0.9, as shown in Figure 5d, the change in the number of sector ring subintervals has a significant effect on the value of the Poincaré plot NDE. The smaller the window width is, the greater the impact is. When the number of sector ring subintervals is below 40, the Poincaré plot NDE shows a significant upward process with the increase in the number, and when it is greater than 40, the Poincaré plot NDE tends to be stable. Therefore, considering the stability of the Poincaré plot NDE and the calculation load, it is suggested that the number of sector ring subintervals is 50. In addition, we can also find that with the increase of Hurst index, the Poincaré plot NDE shows a significant downward trend.

**Figure 6:**
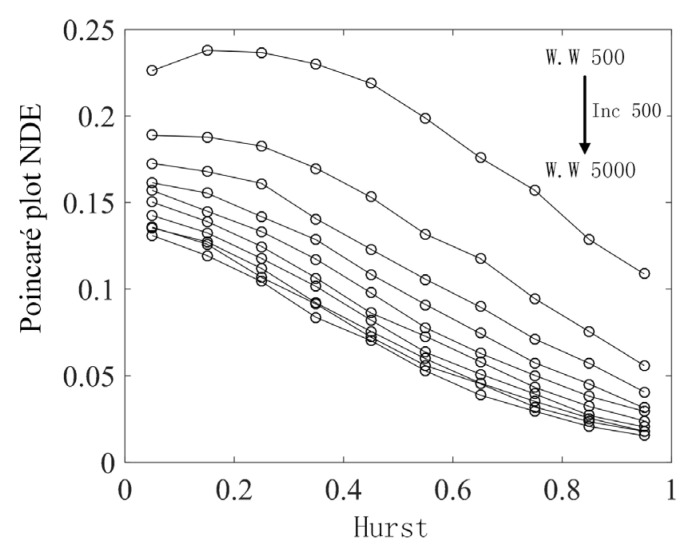
Under different window widths (W.W), the Hurst indices of the fractional Brownian motion time series corresponds to the Poincaré plot NDE.

From the above analysis, it can be seen that the window width has a great impact on the results. After the number of sector ring subintervals is determined, the determination of sliding time window width is particularly important. Here, the optimal window width is determined using a variable window width method, where the value of the window width is varied sequentially in the range [500, 5000] in increments of 500. The objects analyzed are fractional Brownian motion time series with the value of the Hurst index increasing sequentially from 0.05 to 0.9, with each increase of 0.05. Under different window widths, the Poincaré plot NDE method is used to analyze the data in the sliding window, and the average value of all sliding windows in a time series is used as the measurement of that series. Under different window widths, the calculation is repeated 20 times for all time series, and the average value of the 20 results is taken for display. The obtained results are shown in Figure 6. The window width corresponding to each result increases sequentially from top to bottom. It can be found that the analysis results tend to be stable when the window width is greater than 2500. Therefore, the sliding window width for analyzing fractional Brownian motion time series is selected as 2500.

**Figure 7:**
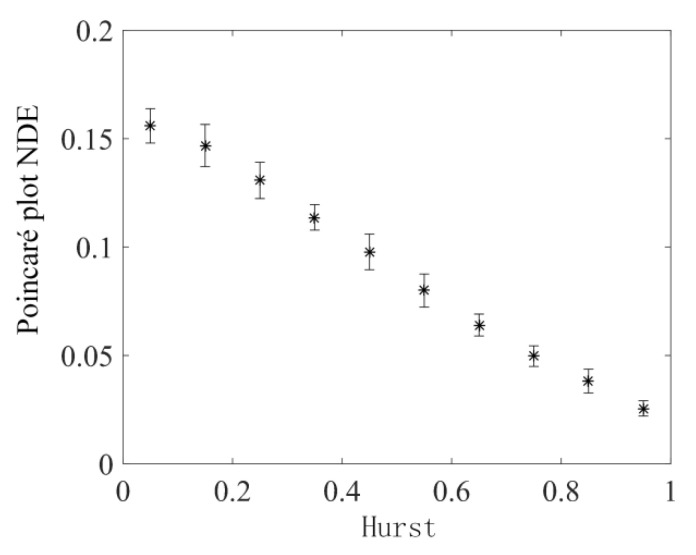
After the parameters are determined, Poincaré plot NDE mean and standard deviation for different Hurst indices corresponding to fractional Brownian motion time series.

After the width of the sliding time window and the number of sector ring subintervals are determined, the fractional Brownian motion time series with different Hurst indices are analyzed using the sliding time window according to the above process using the Poincaré plot NDE, and the moving step is 500 data points. The data analysis process was repeated 30 times, and the results obtained are shown in Figure 7. The Poincaré plot NDE of the fractional Brownian motion time series in Figure 7 is inversely related to the Hurst index, which decreases with the increase in Hurst index. In addition, it can be seen that the standard deviation of the Poincaré plot NDE decreases as Hurst increases. This shows that the proposed method can adequately reflect the dynamic changes in fractional Brownian motion.

### 3.2. Analysis for Sleep EEG

In order to illustrate the performance of the Poincaré plot NDE in real contexts, we selected a publicly available sleep EEG dataset with state labels for analysis.

#### 3.2.1. Description of Sleep EEG Dataset

The analyzed sleep EEG data were obtained from the sleep dataset publicly available on the Internet (30 July 2020, download link: https://www.physionet.org/content/sleep-edf/1.0.0/, accessed on 12 July 2022). The subjects of sleep EEG data collection were Caucasian men and women (21–35 years old) without any drug treatment. The dataset included horizontal EOG, fpz-cz, and pz-oz EEG. The data sampling frequency was 100 Hz. In addition to the artificially annotated hypnogram based on the Rechtschaffen–Kales standard [32], the dataset also contains submental EMG envelopes, nasal airflow, rectal body temperature, and event markers, all sampled at 1 Hz. These non EEG data are not involved in this analysis. In the dataset, the data beginning with 4 “sc” are for non-ambulatory healthy volunteers, and the data beginning with 4 “st” are for subjects with mild difficulty falling asleep but otherwise healthy [33,34]. Sleep stage is divided according to Rechtschaffen–Kales stage standard, specifically divided into AWAKE stage; non-rapid eye movement (NREM) stage 1, stage 2, stage 3, and stage 4; and rapid eye movement (REM) stage, corresponding to the numbers 0, 1, 2, 3, 4, and 5 in the sleep stage map in turn. The exercise time was marked as 6 and the unscored as 9. In 2007, the American Academy of Sleep Medicine (AASM) changed stage 1 and stage 2 to N1 (NREM 1) and N2 (NREM 2), and merged stage 3 and stage 4 into N3 (NREM 3) [35]. The 2.5 h data of the subject sc4002e0 including awake and sleep stages were selected as the analysis object, the electrode was Pz-Oz, the corresponding label position was label 781-1080, and the corresponding data length was 900,000.

#### 3.2.2. Sleep EEG Data Analysis

**Figure 8:**
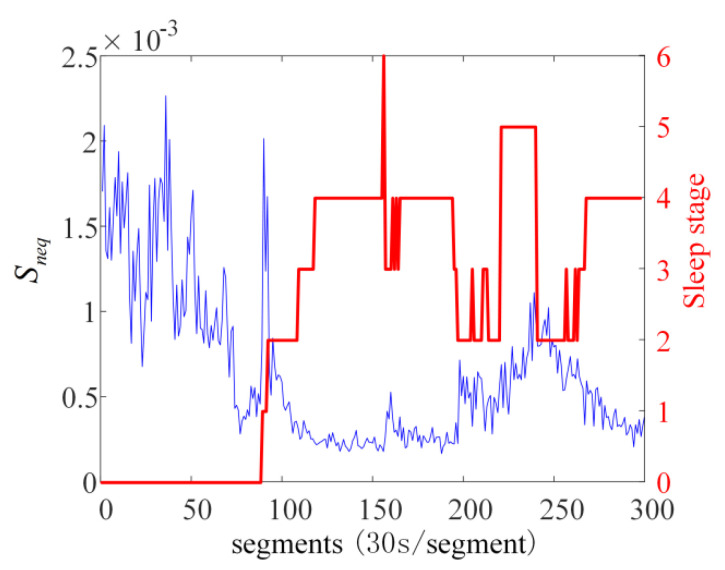
Comparison chart of sleep stage and Poincaré plot NDE analysis results of subjects sc4002e0; red step line is sleep stage and blue curve is Poincaré plot NDE. The red curve indicates different sleep stages.

Sleep EEG signals are susceptible to interference from other physiological signals and the external environment, and the raw data need to be preprocessed to eliminate noise and physiological artifacts before analysis. Firstly, the SVD method is used to remove the trend term of EEG signals. The de-trended EEG signals are bandpass filtered by harmonic wavelet with phase-locked function. The passband range is 1–32 Hz, and the EEG signals to be analyzed are obtained. The preprocessed EEG signals are segmented by 30 s. The sliding window method is used to analyze each segmented data. The window length of the sliding window is 1000 data points, and the step size is 10 data points. The Poincaré plot NDE of each sliding window is calculated, and the average value of the Poincaré plot NDE of all sliding time windows is taken as the value of this segment of data. The analysis results of all segments and corresponding sleep stages are shown in Figure 8.

In Figure 8, the blue curve is a time-varying NDE curve, and the red step line is the sleep stage engraved at the same time with NDE. It can be observed that there is a significant difference between the NDE of EEG during AWAKE stage and sleep stage. The NDE during AWAKE stage is greater than the NDE of sleep stages. The NDE value of N3 stage is smaller than those of N1, N2, and REM stages. The NDE of REM stage is the largest during the entire sleep process. NDE of N1 stage shows a sudden increase pulse shape, and the maintenance time is short. There is an obvious decrease when switching from N1 stage to N2 stage. N1 and N2 stages belong to light sleep state, but there is an obvious difference in NDE. The drastic change of NDE may be the reason why it is difficult to effectively distinguish N1 and N2 stages by using other methods. However, the results of NDE analysis made a clear distinction between N1 and N2 stages. A more meaningful finding is that the method using NDE identified a period before entering N1 when NDE values have a sharp decrease and remain low, but the artificially annotated hypnogram classifies this stage as AWAKE stage, probably because this stage is in a transitional state between wakefulness and sleep, without outward signs of sleep.

**Figure 9:**
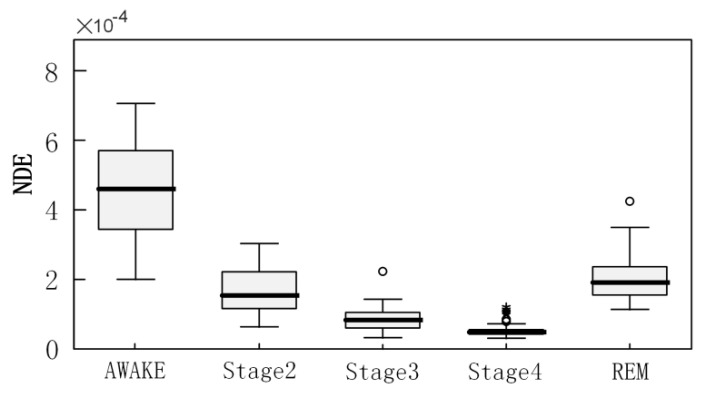
Multi-independent samples nonparametric Jonckheere–Terpstra test boxplots of NDE analysis results for 5 brain states.

To confirm whether there were significant differences in the results of NDE analysis across sleep stages, the segmented EEG data were grouped and statistical significance was applied to these grouped data. The segmented EEG data with the same stage labels were taken as a group. Because the number of segments in stage 1 is too small, the EEG segmented data in state 1 is not considered. Therefore, the EEG segmented data in five states such as AWAKE, stage 2, stage 3, stage 4, and REM were analyzed. To improve the data analysis, EEG segmented data were processed with a sliding time window, the NDE for each sliding time window was calculated, and the average of all sliding window results was taken as the measurement for that segment. Here, the window width of the sliding time window is 1000 data points, and the step size is 10 data points. After the EEG segmented data of all groups were processed, the multi-independent samples non-parametric Jonckheere–Terpstra test was used to test the analysis results. The null hypothesis (H0) was that there was no difference between sleep stages. The significance level was set at *p* = 0.05, and statistical analysis was performed in IBM spss25.0. The Jonckheere–Terpstra test statistic is 4220.5 and the asymptotic significance two-sided test value is 0, reaching a highly significant level, *p* < 0.001, indicating that there is a very significant difference between stages. The boxplots of NDE test results for five brain states are shown in Figure 9.

The results of the pairwise comparison between the stages are shown in Table 1, which presents more detailed information. During pairwise comparison, the null hypothesis is that the NDE values of two stages have the same distribution. The significance level is *p* = 0.05, and the Bonferroni correction has adjusted the significance values for multiple tests. It can be seen that, except for no significant difference between stage 2 and REM (adjusted significance *p* = 0.239), the other paired stags differences all reached a very significant level (adjusted significance *p* < 0.001). These results suggest that NDE has a good discriminative ability for different brain states. For the problem that the difference between stage 2 and REM is not obvious, one reason may be that there are too few EEG data in these two stages; the other reason may be that the experts’ manual annotation error, because there is a significant delay in the conversion of NDE results from S2 to R in Figure 9; and the other possibility is the problem of the method itself, which needs more data for verification.

## 4. Conclusions

The emergence of the Poincaré plot DE solved the problem of nonlinear analysis of time series from an entropy perspective, but showed a lack of differentiation ability when analyzing EEG simulation data, i.e., fractional Brownian motion time series with different Hurst indices. In this paper, by analyzing the reason why the discrimination ability of the Poincaré plot DE is not performed, based on the nonextensive of EEG, taking the distance between each sector ring subinterval and the coordinate origin as the nonextensive parameter, the Poincaré plot NDE suitable for univariate EEG analysis is proposed. The novelty of the method is that the nonextensive parameter of Tsallis nonextensive entropy is extended to a nonextensive parameter set, and different nonextensive parameters are assigned to each structural unit to describe the different effects on the system. At the same time, the nonextensive parameter set is substituted into the NE formula for theoretical derivation, and the constraints of the nonextensive parameters are relaxed to obtain an NNE. Then, the probability density set and the nonextensive parameter set are substituted into the NNE formula to obtain the Poincaré plot NDE.

In order to test the validity of the Poincaré plot NDE, we first applied Poincaré plot NDE to the simulated fractional Brownian motion time series with different Hurst indices. We found that two parameters, the window width and the number of sectoral ring subintervals, had an effect on the Poincaré plot NDE, with the size of the window width having a greater effect and the number of sectoral ring subintervals having a small effect. Once the two parameters were determined, the results of the repeated analysis showed that both the Poincaré plot NDE and their standard deviations decreased with increasing Hurst exponent. The analysis shows that the proposed method is able to accurately measure the dynamics of fractional Brownian motion and has better robustness for time series with large Hurst exponents.

The Poincaré plot NDE is also employed to a publicly available sleep EEG dataset. A 2.5 h segment of EEG data from wakefulness to sleep of subject “sc4002e0” was used for the analysis, which included all stages of sleep. The segmented EEG data were grouped and the multiple independent samples nonparametric Jonckheere–Terpstra test was applied to the Poincaré plot NDE values for these grouped data. Pairwise comparisons showed that the Poincaré plot NDE differences between pairs reached highly significant levels (adjusted significance *p* < 0.001), except for stage2 and REM, where there was no significant difference (adjusted significance *p* = 0.239). The analysis results of the application show that the method incorporates the amplitude information of the signal and has the advantages of ease of calculation, low data length requirements, single input parameter, and high state differentiation capability.

Generally speaking, the reliability of this method is easily affected by the pulse signal with a larger amplitude, which will inhibit the effect of the effective signal on the system, and its disadvantages need to be further studied. As a complementary approach to the evaluation of the EEG, future work will also need to consider the impact of multi-scale factors on the results of the Poincaré plot NDE when analyzing different brain states. In addition, based on the simple calculation characteristics of Poincaré plot NDE, how to realize the online feature extraction of different brain states also needs to be studied. Another shortcoming is that our conclusion is not based on large data analysis, which significantly reduces the reliability of statistical analysis. In the near future, large datasets will be selected to verify the statistical effectiveness of the proposed method, so as to further improve the existing methods.

## Figures and Tables

**Table 1:** Pairwise comparison results of the non-parametric Jonckheere–Terpstra test for multi-independent samples.

Sample 1, Sample 2	Test Statistic	Std. Error	Std. Test Statistic	Significance	Adjusted Significance
Stage 4, Stage 3	506.5	172.724	−5.040	0	0
Stage 4, Stage 2	78.5	281.729	−10.221	0	0
Stag 4, REM	2037	144.600	7.033	0	0
Stage 4, AWAKE	0	254.997	−10.000	0	0
Stage 3, Stage 2	209.5	105.932	−5.414	0	0
Stage 3, REM	514	46.470	5.251	0	0
Stage 3, AWAKE	2	93.673	−7.185	0	0
Stage 2, REM	753	87.387	1.980	0.024	0.239
Stage 2, AWAKE	69	162.301	−8.509	0	0
REM, AWAKE	51	76.920	−5.837	0	0

Each row tests the null hypothesis: sample 1 and sample 2 have the same distribution. The significance level is *p* = 0.05, and the Bonferroni correction has adjusted the significance values for multiple tests.

## Data Availability

The raw data supporting the conclusions of this article will be made available by the authors, without undue reservation, to any qualified researcher.
